# IgM anti-malondialdehyde low density lipoprotein antibody levels indicate coronary heart disease and necrotic core characteristics in the Nordic Diltiazem (NORDIL) study and the Integrated Imaging and Biomarker Study 3 (IBIS-3)

**DOI:** 10.1016/j.ebiom.2018.08.023

**Published:** 2018-08-18

**Authors:** Victor J. van den Berg, Dorian O. Haskard, Artur Fedorowski, Adam Hartley, Isabella Kardys, Mikhail Caga-Anan, K. Martijn Akkerhuis, Rohit M. Oemrawsingh, Robert Jan van Geuns, Peter de Jaegere, Nicolas van Mieghem, Evelyn Regar, Jurgen M.R. Ligthart, Victor A.W.M. Umans, Patrick W. Serruys, Olle Melander, Eric Boersma, Ramzi Y. Khamis

**Affiliations:** a Department of Cardiology, Erasmus MC, Rotterdam, The Netherlands; b National Heart and Lung Institute, Imperial College, London, United Kingdom; c Department of Cardiology, Northwest Clinics, Alkmaar, The Netherlands; d Netherlands Heart Institute (NHI), Utrecht, The Netherlands; e Department of Clinical Sciences, Malmö, Lund University, Clinical Research Center, Malmö, Sweden; f Department of Cardiology, Skåne University Hospital, Malmö, Sweden

**Keywords:** Necrotic core, Lipid core, Oxidized low density lipoprotein, Immunoglobulins, Near-infrared spectroscopy, Intravascular ultrasound

## Abstract

**Background:**

Certain immunoglobulins (Ig) are proposed to have protective functions in atherosclerosis.

**Objectives:**

We tested whether serum levels of IgG and IgM autoantibodies against malondialdehyde low density lipoprotein (MDA-LDL) are associated with clinical coronary heart disease (CHD) and unfavorable plaque characteristics.

**Methods:**

NORDIL was a prospective study investigating adverse cardiovascular outcomes in hypertensive patients. IBIS-3 analyzed lesions in a non-culprit coronary artery with <50% stenosis using radiofrequency intravascular ultrasound (RF-IVUS) and near-infrared spectroscopy (NIRS). Imaging was repeated after a median of 386 days on rosuvastatin. Associations of antibodies with incident CHD and imaging parameters were assessed in the two sub-studies respectively.

**Findings:**

From 10,881 NORDIL patients, 87 had serum sampled at baseline and developed CHD over 4.5 years, matched to 227 controls. Higher titers of IgM anti-MDA-LDL had a protective effect on adverse outcomes, with odds ratio 0.29 (0.11, 0.76; p = 0.012; p = 0.016 for trend). Therefore, the effect was explored at the lesional level in IBIS-3. 143 patients had blood samples and RF-IVUS measurements available, and NIRS was performed in 90 of these. At baseline, IgM anti-MDA-LDL levels had a strong independent inverse relationship with lesional necrotic core volume (p = 0.027) and percentage of plaque occupied by necrotic core (p = 0.011), as well as lipid core burden index (p = 0.024) in the worst 4 mm segment.

**Interpretation:**

Our study supports the hypothesis that lower circulating levels of IgM anti-MDA-LDL are associated with clinical CHD development, and for the first time relates these findings to atherosclerotic plaque characteristics that are linked to vulnerability.

**Research in Context**

**Evidence before this study**

The role of anti-oxidized LDL antibodies in atherosclerosis has been extensively studied. IgM antibodies were hypothesized to be largely protective. There is little data linking antibody levels to plaque characteristics in patients and basic cross-sectional coronary angiography studies have not been conclusive.

**Added value**

This study confirms that high levels of IgM antibodies against oxLDL are protective from coronary heart disease in a nested case control study within a large RCT. In addition, this study shows for the first time, that high levels of these IgM antibodies, and to a lesser extent total serum IgM, are associated with coronary plaque characteristics that reflect plaque stability (i.e. smaller NC and less lipid core on NIRS). These novel findings postulate a mechanistic explanation of how IgM anti-oxLDL antibodies may exert their protective effects in patients with CHD.

**Implications of all the available evidence**

By linking low IgM antibody levels to clinical CHD and unfavorable plaque characteristics, our study may be useful in designing future immunotherapies for the ‘vulnerable plaque’ as well as in focusing patient selection for clinical cardio-protection trials and patient stratification in the clinic.

Alt-text: Unlabelled Box

## 1 Introduction

The immune system exerts both protective and pathogenic effects in atherosclerosis, with a fine balance between maintaining homeostasis and over-activation [1, 2]. Immunoglobulins (Ig) and specific antibodies are relatively stable and easy to quantify, and as such are widely employed as biomarkers. In the pursuit of better prognostic indicators of adverse events due to coronary heart disease (CHD), many groups have studied the role of antibodies against oxidation-specific epitopes on low density lipoprotein (LDL), such as antibodies reacting with phosphorylcholine or malondialdehyde (MDA) [3]. IgM antibodies have mostly been found by in vitro and by preclinical studies to have broadly atheroprotective functions [2, 4, 5]. Furthermore, clinical cardiovascular studies have shown higher IgM anti-MDA-LDL levels are associated with less atherosclerotic burden and better outcomes. In contrast, studies relating IgG antibodies to CHD have been less conclusive [[6], [7], [8], [9], [10], [11], [12], [13], [14], [15]].

In the quest to link the vulnerable patient to the rupture-prone plaque, it is now important to identify factors in the circulation that are related with both incident coronary events and negative characteristics of coronary plaques beyond just degree of arterial narrowing [16, 17]. Although a large necrotic core (NC), thin fibrous cap and a prominent neovasculature are recognized pathological features of plaques most likely to rupture in untreated subjects, few studies have related blood biomarkers to unfavorable plaque characteristics [[17], [18], [19], [20]].

Limited work from randomized control studies as well as cohort studies suggested a link between IgM antibodies against various modifications of LDL and protection from incident cardiovascular events [13]. However, this needs further verification in a high-risk population for CHD, without previously diagnosed cardiovascular disease (CVD). Therefore, we set out to demonstrate this association in a specially-designed nested case control study of the Nordic Diltiazem (NORDIL) study [21], focusing on antibodies against what is considered to be a significant modification of LDL, induced by oxidation, in the form of MDA-LDL.

We first present findings from the NORDIL study [22] confirming the link between IgM anti-MDA-LDL antibodies and protection from CHD. Bearing in mind the unavailability of faithful preclinical models of plaque instability, a major unanswered question is whether levels of IgM anti-MDA-LDL and other antibodies relate to vulnerable plaque characteristics. Using intra-coronary imaging data collected by the Integrated Imaging and Biomarker Study 3 (IBIS-3) [23], we assessed levels of immunoglobulins and anti-MDA-LDL antibodies in relation to detailed information on plaque morphology obtained by radiofrequency intravascular ultrasound (RF-IVUS) and near infra-red spectroscopy (NIRS). We report herein that individuals with low IgM anti-MDA-LDL antibody levels, and to a lesser extent levels of total serum IgM, are significantly more likely to have plaques exhibiting evidence of vulnerability.

Our findings provide substantial support for IgM anti-MDA-LDL antibodies protecting not only from events in NORDIL but also at the level of the plaque in IBIS-3.

## 2 Methods

### 2.1 Study design

#### 2.1.1 NORDIL sub-study

**Fig. 1:**
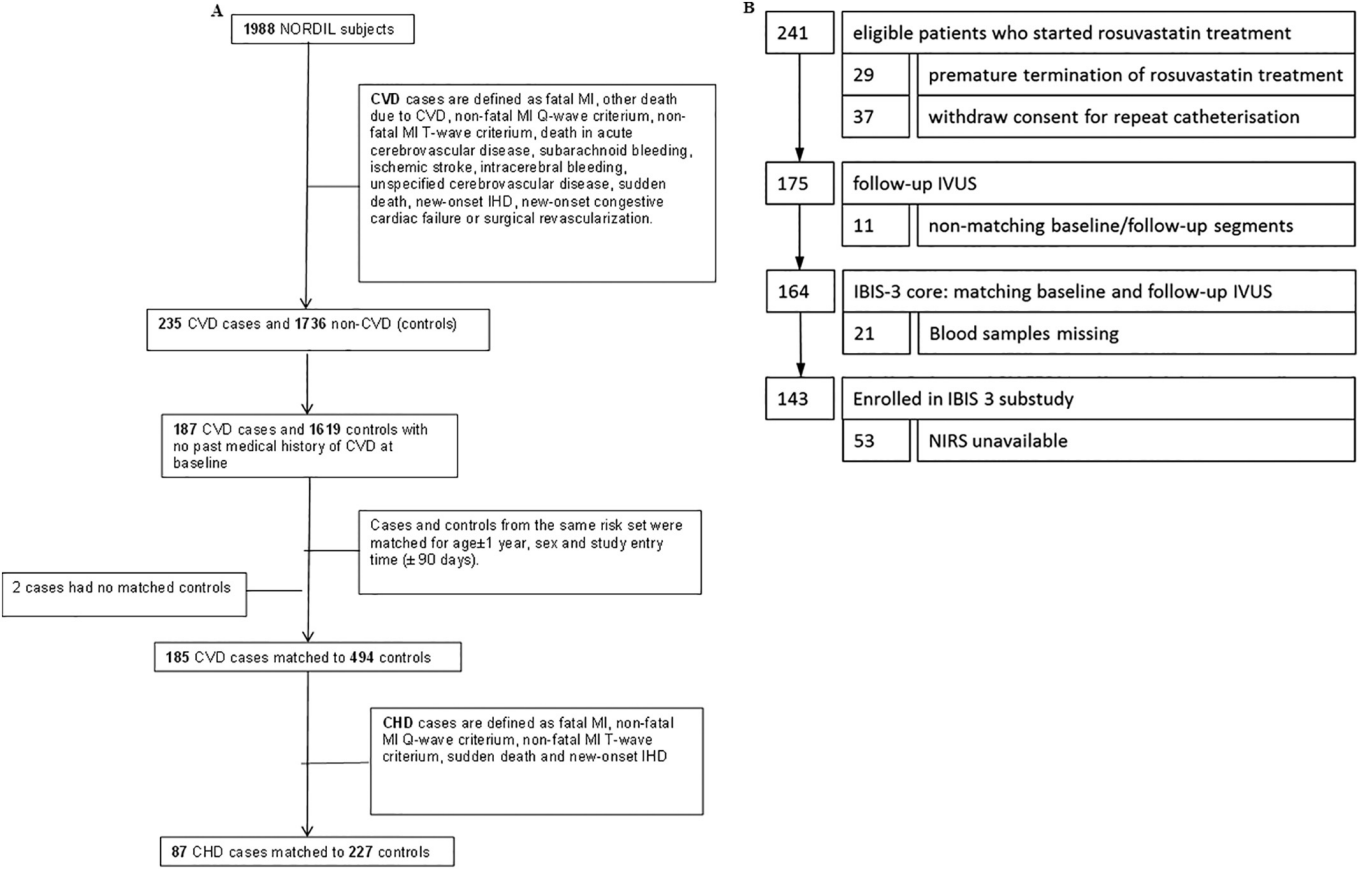
NORDIL and IBIS-3 sub-study Flow Charts. Fig. 1A– NORDIL sub-study flow chart. Fig. 1B – IBIS-3 sub-study flow chart. CVD: cardiovascular disease; CHD: coronary heart disease; MI: myocardial infarction; IHD: ischemic heart disease; IVUS: intravascular ultrasound; NIRS: near-infrared spectroscopy.

The NORDIL study has been extensively described [21, 22]. It is a prospective randomized open trial with blinded endpoint evaluation, designed to assess the effect of diltiazem on cardiovascular outcomes in hypertensive patients versus diuretics, beta-blockers, or both. We designed a nested case control study from the original trial population, with details of the case and control selection shown in the Supplementary Methods Section. 187 CVD cases were identified, of which 88 were classified as coronary heart disease (CHD). Controls were selected from the study population, entered the study before the case was diagnosed with CVD, and were free from CVD themselves. Up to three controls from the same risk-set were matched to each case by age (±1 year), sex and study entry time (±90 days). In total 185 CVD cases were matched to 494 controls, whilst 87 CHD cases were matched to 227 controls (Fig. 1A).

#### 2.1.2 IBIS-3 sub-study

IBIS-3 was a prospective cohort study that was designed to determine the ability of rosuvastatin to decrease NC volume in coronary atherosclerosis. The study was conducted in the Erasmus MC, Rotterdam, the Netherlands from 2010 until 2013, and the population has previously been described in detail [[23], [24], [25]]. The study was approved by the ethics committee of Erasmus MC and all study subjects gave written informed consent. Patients above 18 years of age undergoing diagnostic coronary angiography (CAG) or percutaneous coronary intervention (PCI) for either stable CHD or myocardial infarction (MI) were eligible. After the standard CAG ± PCI, RF-IVUS and NIRS measurements were performed in a non-culprit coronary artery with a diameter stenosis <50%. RF-IVUS and NIRS measurements were performed as per standard protocol. Plaque burden was measured with grayscale IVUS, NC volume by RF-IVUS and the lipid core burden index (LCBI) by NIRS [23]. After a median of 386 days of high dose rosuvastatin treatment (40 mg daily), the measurements of the same segment were repeated. Fig. 1B details the IBIS-3 sub-study patient recruitment.

### 2.2 Biomarkers

Blood samples were drawn at randomization in NORDIL or prior to the procedures in IBIS-3, and were stored at −80 °C. After study completion, samples were transported under controlled conditions to the Vascular Sciences Section at Imperial College, London, UK. We measured total serum IgM and IgG and specific antibodies against MDA-LDL by ELISA, as previously described [7]. Levels of MDA-LDL (a form of oxidized LDL) were assayed using a capture ELISA, using anti-oxLDL monoclonal antibody LO1 [26] for MDA-LDL for capture and goat anti-Apolipoprotein B (ApoB) (Abcam, UK) for biotinylated polyclonal goat anti-ApoB (Abcam, UK) for detection. Secondary detection was with horseradish peroxidase (HRP)-conjugated streptavidin (R&D Systems, Minneapolis, MN) followed by 3,3′,5,5′-tetramethylbenzidine (TMB) (Sigma Aldrich, UK). To measure ApoB levels, we used a capture ELISA with goat-anti ApoB (Abcam, UK) as capture antibody, and detected with the biotiynlated goat anti-ApoB and HRP as above. The plates were read at an optical density of 450 nm using a Synergy HT microplate reader (BioTek, USA). After subtraction of background, the samples were corrected to a reference plasma with a standard curve, and results were expressed in Units (U), as utilized previously [7]. All serological measurements were undertaken by staff blinded to patient characteristics. Quality control and coefficient of variance (CoV) calculations were undertaken and samples retested if they exceeded 5% intra-plate CoV and 15% inter-plate CoV. The intra-plate and inter-plate coefficients of variation (CoV) for all antibody assays and assay ranges are displayed in Supplementary Table 1.

### 2.3 RF-IVUS imaging and measurements

**Fig. 2:**
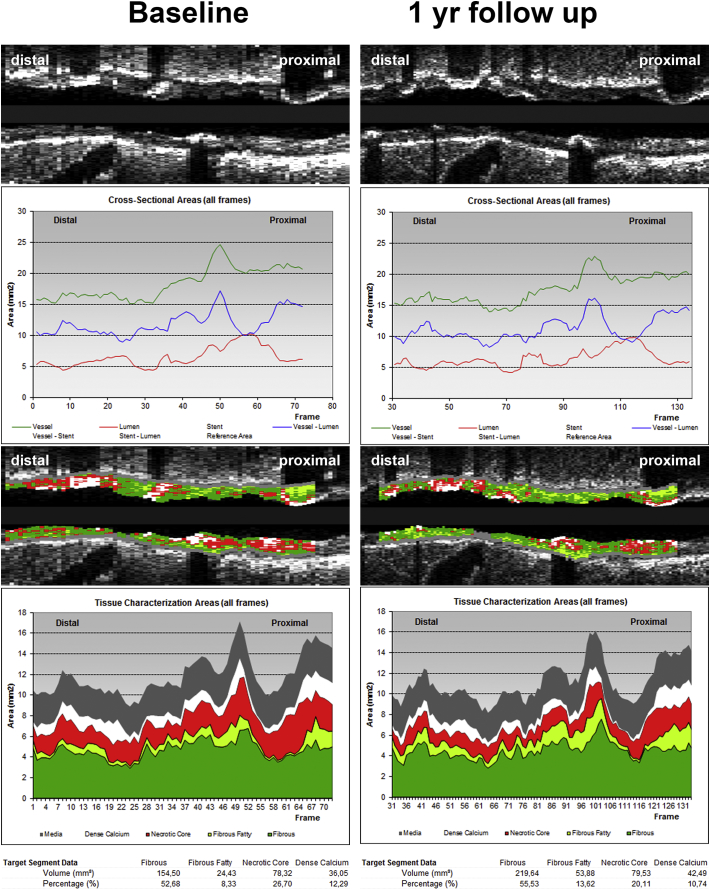
An Example of Baseline and One Year Intravascular Measurements in IBIS-3 (central illustration). IVUS and NIRS analyses (QCU-CMS®, LUMC, Leiden, The Netherlands and IVUS-VH®, Philips Volcano, San Diego, USA, IVUS-NIR, Infraredx®, Burlington, MA, USA) at baseline and at 1 year of the left anterior descending artery of a 51 year old male patient that underwent angioplasty to of the circumflex artery. Panels A and A' show respectively the IVUS grayscale longview of the region of interest (ROI) at baseline and at 1 year follow-up. B and B′ the graphic representation of lumen-, vessel- and plaque areas at baseline and follow-up. Graphs show that there is some decrease in vessel- and plaque size after 1 year without change in lumen size. Panels C and C′ show NIRS, registered with the ROI at baseline and follow-up. The yellow areas indicate lipid rich plaque. The highest LCBI in 4 mm is located between the lines and shows at follow-up a slight decrease. Panels D, E, D' and E' show the VH-analyses of the ROI at baseline and follow-up, showing a significant decrease of necrotic core (in red, 26,7 to 20,1%) after 1 year, mostly in favor of fibrous fatty plaque (in light green) which increased from 8,3 to 13,6% of the total plaque volume. (Illustration: Jurgen M.R. Ligthart, RT; Karen Th. Witberg, CCRN).

20-MHz IVUS catheters (Eagle-Eye; Volcano Corporation, San Diego, CA, USA) were used at a continuous motorized pullback speed of 0.5 mm/s (R-100 pullback device; Volcano Corporation). The IVUS images were analyzed offline for plaque burden and NC volume by an independent core laboratory (Cardialysis BV, Rotterdam, the Netherlands), blinded for clinical and biomarker data. The IVUS grayscale and IVUS radiofrequency analyses, also known as IVUS virtual histology (IVUS-VH), were performed using pcVH 2.1 and qVH (Volcano Corp., San Diego, CA) software. The external elastic membrane and luminal borders were contoured for each frame (median inter-slice distance, 0.40 mm). Extent and phenotype of the atherosclerotic plaque were assessed. Plaque volume was defined as the total volume of the external elastic membrane occupied by atheroma [23]. Plaque burden was defined as plaque and media cross-sectional area divided by external elastic membrane cross-sectional area and is presented as a percentage. Briefly, frames corresponding to the R wave on the ECG were selected. These images were analyzed using both grayscale as well as the RF virtual histology and volumes calculated automatically. Following characterization of the composition of the atherosclerotic plaque, the percentage and total volume of the NC were determined [23, 27]. Fig. 2 demonstrates an example of IVUS-VH measurements at baseline and 1 year.

### 2.4 NIRS

NIRS was performed with the Infraredx system (Infraredx, Burlington, MA, USA), at a pullback speed of 0.5 mm/s. The NIRS-system used acquires 1000 measurements per 12.5 mm of coronary artery of 1 to 2 mm^2^ from a depth of approximately 1 mm^2^ perpendicular to the long axis of the catheter [28]. The coronary composition is visualized in a chemogram in which a lipid core is displayed as yellow. The LCBI is a score from 0 to 1000 that reflects the amount of yellow present on the chemogram. We assessed the LCBI of the entire region of interest, and of the 10 mm and 4 mm segments with the highest LCBI [29, 30]. Both the 10 and 4 mm segments that were measured at follow-up corresponded to the exact segment of artery that had the worst 4 or 10 mm measured at baseline.

### 2.5 Statistical analyses

Continuous variables are presented as mean ± standard deviation (SD) or median ± interquartile range (IQR), depending on the normality of the distribution. Categorical variables are presented as numbers and percentages. Analyses were performed with R statistical software (version 3.3.1, available at: www.r-project.org), SAS V9.4 (SAS Institute, Cary, NC, USA), or STATA V12 (STATA Corporation, College Station, TX, USA). Two-tailed p values of <0.05 were considered statistically significant. Further details on statistical methodology are provided in the Supplementary Methods.

## 3 Results

### 3.1 NORDIL sub-study baseline characteristics

The NORDIL study enrolled 10,881 hypertensive patients, of which 1988 samples were available for analysis. 235 cases with CVD were identified, of which 187 had no history of CVD at baseline. 88 cases of CHD were identified. In total, after samples with no matched controls and insufficient sera available were removed, 185 CVD cases were matched to 494 controls, and 87 CHD cases were matched to 227 controls. Baseline characteristics can be seen in Supplementary Table 2. Mean age was 60·79 (6.37) versus 61·25 (6.30) years in the cases and controls respectively (59.3% versus 61.6% male). There were no significant differences between the groups, with the exception of smoking rate (p < 0·0001), HDL-cholesterol (p = 0·04) and diabetes (p = 0·021).

### 3.2 IgM anti-MDA-LDL antibodies confer protection from clinical CHD in NORDIL

There was a significantly higher median level of IgM anti-MDA-LDL in the controls (0·85 (0·55, 1·20) units) versus the CVD cases (0·76 (0·54, 1·04) units; p = 0·039) at baseline, whereas no relationship was demonstrated with IgG anti-MDA-LDL (p = 0·38) (Supplementary Table 3).

As CVD may have a heterogeneous causal pathology, we focused on the relationship of antibodies against MDA-LDL with incident CHD events. After controlling for variables with significant inter-group variation (smoking status, diabetic status, baseline HDL) and randomized blood pressure treatments, as well as total IgG and IgM, immunoglobulin levels were log-transformed and divided into tertiles. The highest tertile of IgM anti-MDA-LDL was found to have a substantial protective effect on the development of CHD, with odds ratio (OR) 0·29 (0·11, 0·76; p = 0·012; p = 0·016 for trend) (Table 1). No such relationship was found with IgG anti-MDA-LDL antibodies.

**Table 1:** Odds ratios of Coronary Heart Disease per tertile of baseline antibodies from the NORDIL sub-study.

Parameter	Cases/Controls	OR (95% CI)	P value
Per 1 SD increase in log_e_ IgG	87/227	1.00 (0.74, 1.35)	1.00
Tertiles:			
0.51–0.91	30/74	1.00 (Ref)	
0.92–1.18	24/81	0.65 (0.33, 1.28)	0.22
1.18–2.53	33/72	0.93 (0.45, 1.92)	0.84
Trend		p = 0.82	

Per 1 SD increase in log_e_ IgM	87/227	0.70 (0.46, 1.06)	0.09
Tertiles:			
0.18–0.62	31/73	1.00 (Ref)	
0.62–1.07	35/70	0.90 (0.45, 1.80)	0.76
1.08–3.16	21/84	0.29 (0.11, 0.76)	0.012
Trend		p = 0.016	

Odds Ratios of Coronary Heart Disease in Relation to Baseline IgG and IgM Anti-MDA-LDL Antibodies (Per Standard Deviation Increase in Log-transformed Antibodies and in Antibodies Tertiles) from the NORDIL sub-study.

Adjusted for significant differences between cases and controls (smoking status, diabetic status, baseline HDL, randomized blood pressure treatments plus either total IgG or total IgM. Ig: immunoglobulin; HDL: high-density lipoprotein; MDA-LDL: malondialdehyde-modified low density lipoprotein; OR: odds ratios; CI: confidence intervals; SD: standard deviation.

### 3.3 IBIS-3 sub-study baseline characteristics

We examined samples from IBIS-3 to explore the putative protective effect of IgM anti-MDA-LDL antibodies at the level of the atherosclerotic plaque. Of the 241 patients eligible to start rosuvastatin treatment, 143 had both blood samples and matching baseline and follow-up RF-IVUS measurements available (Fig. 1B). The present sub-study was representative of the parent IBIS-3 study, with no differences in any of the patient characteristics (Table 2). The mean age of participants was 59·6 (9·0) years and 84·6% of recruits were male. The study population had extensive risk factors for CHD, including diabetes (28%), hypertension (89%) and hypercholesterolemia (90%). 41% were current smokers and 5% had documented renal failure.

**Table 2:** Baseline characteristics of the IBIS-3 sub-study population.

	IBIS-3 core:	IBIS-3 sub-study:	P Value
	(n = 164)	markers (n = 143)	
Age (SD)	59.8 (9.0)	59.6 (9.0)	0.44
Gender (% men)	138 (84.1)	121 (84.6)	0.67
BMI* (SD)	28.6 (4.4)	28.8 (4.5)	0.12
Statin use (SD)	156 (95.1)	135 (94.4)	0.60

Cardiovascular risk factor (%)			
Diabetes	34 (20.7)	28 (19.6)	0.90
Hypertension	104 (64.2)	89 (62.7)	0.28
Hypercholesterolemia ±	103 (63.6)	90 (63.4)	0.89
Current smoker	46 (28.0)	41 (28.7)	0.64
Family history of CAD±	89 (54.6)	76 (53.5)	0.47
Renal failure	6 (3.7)	5 (3.5)	0.57

History of cardiovascular disease (%)			
MI	49 (29.9)	42 (29.4)	0.71
CABG	1 (0.6)	0 (0)	0.13
PCI	59 (36.0)	51 (35.7)	0.83
Stroke	15 (9.1)	14 (9.8)	0.70

Indication for coronary angiography (%)			
STEMI	24 (14.7)	21 (14.7)	0.44
NSTE ACS	44 (26.8)	36 (25.2)	
Stable angina	96 (58.5)	86 (60.1)	

PCI performed (%)	146 (89.0)	128 (89.5)	0.71
Time between procedures (IQR, days)	386 (372–404)	386 (374–403)	0.72
Last used Rosuvastatin dose (IQR, mg)	40 (20–40)	40 (20–40)	0.93

IBIS 3 core: patients with completed treatment phase and matching baseline and follow-up RF-IVUS; IBIS-3 substudy: patients with available blood samples for measuring oxLDL;

* missing in 10 patients (both cohorts); missing in 2 patients and one patient respectively; ± missing in 1 patient (both cohorts); RF-IVUS: radiofrequency intravascular ultrasound; oxLDL: oxidized low density lipoprotein; BMI: body mass index; CAD: coronary artery disease; MI: myocardial infarction; CABG: coronary artery bypass grafting; PCI: percutaneous coronary intervention; STEMI: ST-elevation myocardial infarction; NSTE ACS; non-ST-elevation acute coronary syndrome. P values derived from testing the differences between patients included in sub-cohort (n = 143) and those not included (n = 21), using Chi-square tests and Fisher's Exact tests for categorical variables and *t*-tests and Mann–Whitney *U* tests for normally and non-normally distributed continuous variables respectively.

### 3.4 Baseline IgM anti-MDA-LDL antibodies indicate RF-IVUS-derived necrotic core characteristics

At baseline, IgM anti-MDA-LDL antibody levels had a strong inverse relationship with lesional NC volume (p = 0·027) and percentage (p = 0·011) (Table 3). Thus, the NC volume associated with the lowest quartile of IgM anti-MDA-LDL antibodies was more than twice that associated with the highest quartile. This relationship survived correction for age, sex, diabetes, smoking and previous use of statins (Table 3, Trend Model 1). It also survived correction for both HDL- and LDL-cholesterol levels (Table 3, trend Model 2). The relationship was however partially dependent on total serum IgM, as lower IgM levels also reflected an unfavorable NC percentage on RF-IVUS (Table 3, Model 3). However, IgM anti-MDA-LDL antibody levels still predicted NC percentage after correction for all variables (Table 3, Model 3). There was no correlation between IgG antibodies, total serum IgG, HDL-cholesterol, LDL-cholesterol, ApoB or oxLDL with any of the imaging parameters (Table 3 and Supplementary Table 4).

**Table 3:** Baseline RF-IVUS measurements per quartile of baseline antibodies from IBIS-3 sub-study.

Biomarker		Plaque Volume	Plaque Burden	NC Volume	NC Percentage
		Median mm3 (IQR)	Median % (IQR)	Median mm3 (IQR)	Median % (IQR)
IgM anti-MDA-LDL antibodies	Lowest	235.4 (205.6, 330.3)	44.0 (37.3, 49.3)	25.8 (15.7, 44.0)	21.0 (18.2, 25.5)
	Second	202.8 (140.2, 294.8)	41.6 (32.3, 50.0)	22.4 (6.1, 47.2)	21.3 (17.6, 23.9)
	Third	212.1 (143.2, 272.7)	38.8 (33.7, 47.2)	18.8 (7.7, 33.7)	19.9 (14.9, 24.0)
	Highest	188.8 (150.3, 225.8)	36.7 (31.9, 45.9)	11.5 (5.7, 22.8)	17.6 (12.1, 22.5)
Trend Model 1		p = 0.10	p = 0.14	p = 0.027	p = 0.011
Trend Model 2		p = 0.093	p = 0.15	p = 0.024	p = 0.0074
Trend Model 3		p = 0.13	p = 0.38	p = 0.060	p = 0.044
Total Serum IgM	Lowest	235.8 (168.9, 304.4)	41.8 (33.1, 45.3)	21.4 (8.9, 38.7)	19.8 (17.3, 25.1)
	Second	206.0 (169.0, 342.2)	45.2 (37.4, 49.0)	23.8 (14.2, 59.0)	21.3 (18.6, 25.0)
	Third	189.9 (136, 279.3)	38.2 (32.1, 52.5)	18.7 (6.9, 29.3)	19.7 (13.7, 26.1)
	Highest	206.7 (150.7, 246.1)	36.7 (31.5, 45.9)	13.9 (6.5, 23.1)	17.7 (12.5, 22.1)
Trend Model 1		p = 0.56	p = 0.43	p = 0.12	p = 0.0071
Trend Model 2		p = 0.51	p = 0.36	p = 0.10	p = 0.0059
IgG anti-MDA-LDL antibodies	Lowest	220.3 (139.5, 328.6)	38.6 (32.7, 47.8)	21.2 (8.4, 44.6)	20.3 (17.5, 27.2)
	Second	230.1 (175.2, 293.8)	43.0 (34.0, 48.4)	20.3 (13.3, 33.5)	20.2 (15.0, 23.0)
	Third	200.8 (147.5, 239.4)	38.2 (32.8, 45.6)	15.7 (6.5, 26.9)	19.4 (14.2, 23.2)
	Highest	212.4 (159.6, 334)	42.9 (33.1, 49.5)	20.2 (7.3, 44.0)	20.3 (14.8, 24.9)
Trend Model 1		p = 0.69	p = 0.62	p = 0.38	p = 0.34
Trend Model 2		p = 0.76	p = 0.52	p = 0.44	p = 0.31
Trend Model 3		p = 0.56	p = 0.70	p = 0.32	p = 0.39
Total Serum IgG	Lowest	201.2 (144.3, 265.1)	38.9 (32.8, 47.0)	17.7 (8.4, 27.0)	19.5 (17.0–22.6)
	Second	211.6 (145.5, 304.2)	42.1 (32.3, 46.1)	15.2 (7.4, 41.9)	19.8 (14.4, 25.9)
	Third	245.9 (170.7, 373.6)	42.8 (34.5, 51.0)	23.1 (11.5, 44.4)	20.9 (16.2, 23.6)
	Highest	209.8 (148.0, 301.0)	41.1 (28.0, 47.8)	19.1 (4.6, 38.8)	20.0 (12.7, 25.1)
Trend Model 1		p = 0.69	p = 0.88	p = 0.85	p = 0.77
Trend Model 2		p = 0.69	p = 0.89	p = 0.87	p = 0.76
LDL-Cholesterol	Lowest	206.0 (136.3, 290.4)	37.3 (30.0, 46.7)	15.8 (6.5, 33.2)	19.8 (15.0, 22.4)
	Second	245.9 (165.4, 327.4)	43.8 (34.3, 48.8)	22.7 (8.9, 57.1)	22.0 (18.2, 26.6)
	Third	216.4 (146.6, 299.5)	43.1 (33.6, 48.7)	20.3 (6.5, 36.0)	19.7 (14.8, 24.0)
	Highest	200.8 (150.0, 253.4)	40.1 (33.8, 45.3)	15.7 (9.3, 23.0)	18.6 (13.5, 24.0)
Trend Model 1		p = 0.91	p = 0.63	p = 0.68	p = 0.19
HDL-Cholesterol	Lowest	210.9 (163.2, 246.6)	41.8 (33.0, 47.9)	19.8 (8.0, 27.4)	19.8 (16.4, 22.5)
	Second	212.6 (123.0, 333.3)	41.4 (32.5, 49.2)	15.6 (5.4, 55.1)	19.4 (14.2, 23.2)
	Third	204.0 (141.5, 268.3)	37.2 (32.6, 45.0)	16.0 (7.7, 32.1)	22.3 (17.5, 25.6)
	Highest	229.3 (168.8, 330.6)	43.1 (35.8, 48.1)	21.7 (10.8, 35.8)	18.9 (14.6, 24.4)
Trend Model 1		p = 0.26	p = 0.74	p = 0.37	p = 0.76

P values based on a linear trend test across the four quartiles of the antibodies in a linear regression model,

Model 1: adjustment for age, sex, diabetes, smoking, and previous use of statins.

Model 2: additional adjustment for LDL and HDL-cholesterol.

Model 3: Model 2 plus adjustments for either total IgG or IgM.

RF-IVUS volumes are standardized for the measured segment length by dividing volume through segment length and then multiplication by the median segment length. HDL was missing in two cases, therefore, the results of model 2, 3 and the results of HDL itself are based on 141 patients.

Immunoglobulin and specific antibody percentile levels in Units (U) as measured by ELISA (based on OD450) and (in g/L as interpolated per standard curves for total Ig levels) were: total IgG: 25th centile 0.97 (8.17 g/L), 50th centile 1.09 (10.71 g/L), 75th centile 1.23(13.08 g/L); Total IgM: 25th centile 0.89 (0.65 g/L), 50th centile 1.21 (1.05 g/L), 75th centile 1.49 (1.49 g/L); IgG anti-MDA-LDL 25th centile 0.30, 50th centile 0.39, 75th centile 0.55; IgM anti-MDA-LDL 25th centile 0.76, 50th centile 1.18, 75th centile 1.64. mm^3^: cubic millimeter; IQR: interquartile range; NC: necrotic core tissue; Ig: immunoglobulins; MDA-LDL: malondialdehyde-modified low density lipoprotein; HDL: high-density lipoprotein.

### 3.5 IgM anti-MDA-LDL antibodies predict lipid core burden index at the 4-mm maximal segment at baseline

In addition to the inverse relationship with lipid core volume and percentage measured by RF-IVUS, IgM anti-MDA-LDL antibodies were also able to predict the LCBI in the worst affected 4 mm section (maxLCBI_4mm_) in the non-culprit coronary artery segment using NIRS. The median maxLCBI_4mm_ NIRS score was 308 (183·8, 355·0) in the lowest quartile of antibody levels, whilst those with the highest antibody levels had a much lower score (less than half) of 133·0 (22·5, 303·5), p = 0·024 for trend (Table 4). This relationship also survived correction for age, sex, diabetes, smoking, and previous use of statins (Table 4, Model 1) as well as HDL and LDL levels (Table 4, Model 2). However, whilst total serum IgM was not significantly related to maxLCBI_4mm_, adjustment for total serum IgM removed statistical significance for the inverse relationship between maxLCBI_4mm_ and IgM anti-MDA-LDL antibodies (Table 4, Model 3). There was a similar relationship between IgM anti-MDA-LDL and the larger LCBI regions of interest however these did not reach statistical significance. Neither IgG antibodies, LDL- or HDL-cholesterol, ApoB or oxLDL predicted any of the NIRS-derived parameters (Table 4 and Supplementary Table 4).

**Table 4:** Baseline LCBI measured by NIRS per quartile of baseline antibodies, low density lipoprotein and high density lipoprotein from the IBIS-3 sub-study.

Biomarker		LCBI full region of interest	LCBI max 10 mm	LCBI max 4 mm
		Median score (IQR)	Median score (IQR)	Median score (IQR)
IgM anti-MDA-LDL antibodies	Lowest	56.0 (33.5, 70.5)	172.5 (118.2, 234.8)	308.0 (183.8, 355.0)
	Second	15.0 (0.25, 49.8)	51.0 (0.5, 156.8)	114.5 (1.5, 264.8)
	Third	41.5 (7.8, 70.5)	87.0 (361.0, 207.0)	155.5 (73.3, 301.8)
	Highest	22.0 (5.0, 56.0)	86.0 (9.5, 161.5)	133.0 (22.5, 303.5)
Trend Model 1		P = 0.29	P = 0.14	P = 0.024
Trend Model 2		P = 0.29	P = 0.15	P = 0.024
Trend Model 3		P = 0.22	P = 0.25	P = 0.11
Total Serum IgM	Lowest	40.0 (21.5, 61.0)	148.0 (59.5, 191.0)	230.0 (132.5, 345.5)
	Second	42.5 (9.75, 61.75)	139 (29.0, 209.0)	253.0 (67.0, 337.0)
	Third	17.5 (0.0, 66.75)	74.0 (0.0, 160.2)	128.0 (0.0, 306.5)
	Highest	29.0 (6.0, 71.5)	103.0 (26.5, 186.0)	165.0 (59.5, 303.5)
Trend Model 1		P = 0.89	P = 0.34	P = 0.12
Trend Model 2		P = 0.89	P = 0.36	P = 0.10
IgG anti-MDA-LDL antibodies	Lowest	27.0 (14.5, 61.0)	100.0 (54.8, 194.5)	193.5 (125.5, 338.5)
	Second	45.5 (27.5, 71.5)	157.5 (55.5, 207.8)	268.5 (131.2, 331.2)
	Third	17.0 (0.0, 60.5)	62.0 (0.0, 145.0)	96.0 (0.0, 229.2)
	Highest	41.0 (4.0, 76.5)	118.0 (17.0, 245.5)	165.0 (42.0, 325.5)
Trend Model 1		P = 0.93	P = 0.62	P = 0.29
Trend Model 2		P = 0.94	P = 0.59	P = 0.28
Trend Model 3		P = 0.50	P = 0.30	P = 0.14
Total Serum IgG	Lowest	27.0 (11.0–61.5)	65.0 (28.5, 171.5)	143.0 (64.0, 292.5)
	Second	33.0 (11.8, 59.8)	129.0 (34.5, 201.2)	217.5 (82.3, 339.5)
	Third	25.5 (2.0, 60.3)	83.0 (5.5, 163.5)	166.0 (14.8, 333.5)
	Highest	45.0 (12.5, 84.5)	153.5 (76.5, 229.8)	216.5 (109.8, 331.2)
Trend Model 1		P = 0.35	P = 0.55	P = 0.66
Trend Model 2		P = 0.36	P = 0.54	P = 0.68
LDL-Cholesterol	Lowest	27.0 (3.5, 64.0)	75.0 (9.0, 187.0)	183.0 (22.5, 284.5)
	Second	42.0 (24.0, 61.0)	134.0 (59.0, 225.8)	206.0 (124.8, 348.2)
	Third	23.5 (2.0, 68.0)	107.0 (4.0, 204.0)	159.0 (11.0, 319.0)
	Highest	42.5 (8.5, 76.5)	119.0 (28.5, 173.8)	193.5 (71.8, 332.0)
Trend Model 1		P = 0.63	P = 0.58	P = 0.88
HDL-Cholesterol	Lowest	37.0 (7.5, 66.0)	148.0 (28.0, 195.0)	255.0 (66.0, 336.5)
	Second	30.0 (5.3, 46.0)	78.0 (9.3, 133.0)	139.5 (20.8, 251.0)
	Third	28.0 (7.5, 69.8)	103.0 (27.0, 234.0)	136.0 (60.0, 342.0)
	Highest	34.0 (18.5, 70.3)	150.0 (55.3, 212.8)	237.0 (88.3, 328.8)
Trend Model 1		P = 0.93	P = 0.48	P = 0.97

P values based on a linear trend test across the four quartiles of the antibodies in a linear regression model.

Model 1: adjustment for age, se x, diabetes, smoking, and previous use of statins.

Model 2: additional adjustment for LDL and HDL-cholesterol.

Model 3: model 2 plus either total IgG or IgM.

LCBI: lipid core burden index; IQR: interquartile range; NC: necrotic core tissue; Ig: immunoglobulins; MDA-LDL: Malondialdehyde-modified low density lipoprotein; HDL: high-density lipoprotein.

During the analyses of the association between the immunoglobulins with LCBI full region of interest and LCBI worst 10 mm, a major outlier with a positive effect on the association was identified. To ensure the validity of our results and to prevent violation of the assumption of the linear regression models, this patient was discarded from the analyses.

HDL was missing in two cases, therefore, the results of model 2, 3 and the results of HDL itself are based on 88 patients.

Limits of immunoglobulin and specific antibody quartiles are as in Table 3.

### 3.6 Changes in parameters after treatment with rosuvastatin for one year

A comparison between baseline and follow-up data for RF-IVUS and biomarker variables is shown in Table 5. As expected, LDL was significantly reduced and HDL significantly increased by −0·76 mmol/L (−0·92, −0·59; p < 0·001) and 0·13 mmol/L (0·08, 0·17; p < 0·001) respectively in patients after one year of rosuvastatin treatment. Despite this, and as observed in the main IBIS-3 study [23], there was an overall progression in grayscale IVUS measured plaque volume over the one year study period, with a mean increase of 4·79 (0·23, 9·34) mm^3^. However, as there was no change in the gross NC volume, there was a decrease in NC percentage of −1·25% (−2·29, −0·21) (Table 5). There were also no significant changes seen in NIRS parameters. Interestingly, mean total IgM dropped over one year by 0·16 g/L (−0·27, −0·05; p = 0·004). There was a trend towards reduction in the IgM anti-MDA-LDL antibodies, but this was not statistically significant. Total serum IgG and IgG anti-MDA-MDL antibodies were not affected over the same time period (Table 5). None of the baseline immunoglobulin, antibody or lipid levels were able to predict imaging changes upon follow-up (Supplementary Tables 5 and 6).

**Table 5:** Baseline and follow-up RF-IVUS imaging, NIRS measurements, and immunoglobulin levels from the IBIS-3 sub-study.

	Baseline		Follow-up		Change	
	Mean (SD)	Median (IQR)	Mean (SD)	Median (IQR)	Mean (95% CI)	p value

Procedural						
Plaque volume, mm3	241.6 (149.8)	203.3 (143.6, 304.4)	246.4 (147.9)	208.6 (146.5, 299.7)	4.79 (0.23, 9.34)	0.040
Plaque Burden, %	40.3 (10.2)	40.5 (32.9, 47.8)	41.3 (9.7)	40.9 (33.5, 49.7)	0.95 (0.35, 1.55)	0.002
NC volume, mm3	28.1 (31.0)	17.2 (7.5, 36.7)	27.1 (30.8)	19.1 (6.2, 32.5)	−1.00 (−2.65, 0.64)	0.230
NC percentage, %	20.0 (8.1)	19.9 (15.2, 24.9)	18.9 (7.1)	19.6 (14.8, 23.8)	−1.25 (−2.29, −0.21)	0.019
LCBI full ROI	45.0 (51.4)	33.5 (7, 66.5)	46.8 (39.6)	41.5 (10.25, 75)	−1.7 (−12.6, 9.1)	0.751
LCBI worst 10 mm	130.0 (121.1)	108 (27, 201)	135.0 (111.3)	126 (47, 198)	−5.0 (−26.9, 16.9)	0.652
LCBI worst 4 mm	202.9 (162.2)	182 (63, 332)	214.1 (148.9)	203 (89, 325)	−11.2 (−40.2, 17.7)	0.443

Biomarker						
IgM anti-MDA-LDL antibodies, Units	1.27 (0.61)	1.18 (0.77, 1.62)	1.22 (0.58)	1.19 (0.80, 1.52)	−0.07 (−0.15, 0.01)	0.092
Total IgM, g/L	1.26 (1.03)	1.06 (0.65, 1.58)	1.10 (0.75)	0.93 (0.62, 1.34)	−0.16 (−0.27, −0.05)	0.004
IgG anti-MDA-LDL antibodies, Units	0.45 (0.39)	0.39 (0.30, 0.54)	0.47 (0.22)	0.42 (0.30, 0.62)	0.06 (−0.01, 0.14)	0.104
Total IgG, g/L	11.2 (4.7)	10.7 (8.3, 13.1)	10.5 (4.7)	10.1 (7.6, 12.6)	−0.66 (−1.57, 0.26)	0.159
LDL-cholesterol, mmol/L	2.51 (0.87)	2.37 (1.92, 3.00)	1.75 (0.72)	1.61 (1.28, 2.02)	−0.76 (−0.92, −0.59)	<0.001
HDL-cholesterol, mmol/L^a^	1.12 (0.31)	1.08 (0.93, 1.30)	1.25 (0.37)	1.22 (0.99, 1.46)	0.13 (0.08, 0.17)	<0.001

P values are based on linear mixed models (with patients as random intercept) to test if change is different from 0; Antibodies against MDA-LDL are log-transformed with base number 2.

SD: standard deviation; IQR: interquartile range; CI: confidence interval; mm^3^: cubic millimeter; NC: necrotic core tissue; LCBI: lipid core burden index; ROI: region of interest; Ig: immunoglobulins; g/L: grams per liter; MDA-LDL: malondialdehyde-modified low density lipoprotein; HDL: high-density lipoprotein; mmol/L: millimoles per liter.

a Missing in 2 patients.

## 4 Discussion

Although several studies have reported links between higher serum IgM anti-oxLDL antibody level and lower incidence of CHD, as yet we have very little knowledge of whether high levels of these antibodies relate to different plaque characteristics, or whether protection is by some other means. We have provided this link herein, first demonstrating that higher levels of IgM anti-MDA-LDL independently confer protection from CHD in a well-characterized clinical endpoint-driven population, and secondly, connecting low IgM anti-MDA-LDL antibody levels with unfavorable plaque morphology in a well-characterized intravascular coronary imaging study.

The NORDIL sub-study confirms the protective effect of IgM anti-MDA-LDL on clinical CHD, which has been recognized previously [[6], [7], [8], 13, 31]. However, apart from a few studies [7, 8], most of the evidence is derived from general populations, rather than from clinical endpoint driven studies in high-risk individuals. As such, the findings from this NORDIL sub-study, in a hypertensive population, are very useful.

Moreover, we have demonstrated here for the first time, a direct inverse association between anti-MDA-LDL antibodies and intravascular imaging (both IVUS and NIRS)-derived unfavorable coronary plaque characteristics. Our ability to make this association was heavily dependent on the sophisticated intracoronary imaging techniques used for plaque characterization. Previous work using conventional angiography did not find a relationship between anti-oxLDL antibodies and overall disease burden [8], although there are some studies reporting a link with degree of angiographic stenosis [32]. In keeping with this, we did not find a relationship between antibody levels and the total plaque burden measured by grayscale IVUS. Rather, the salient findings of this study concern more precise characteristics of plaque vulnerability related to the lipid core. The prognostic relevance of assessing non-culprit lesions is strongly supported by recent studies in which larger lipid rich non-culprit plaques were associated with higher risk of future cardiovascular events [25, 27, 33].

Our most striking significant finding was that low levels of IgM anti-MDA-LDL antibodies were associated with greater coronary NC volume and lipid core burden of the worst affected 4 mm segment. Surprisingly, the antibodies performed far better than lipids in this respect, as levels of HDL- and LDL-cholesterol, ApoB and oxLDL did not relate significantly to plaque characteristics. Furthermore, the significance of associations for IgM anti-MDA antibodies was not affected by adjusting the data for levels of LDL- and HDL-cholesterol.

The inverse baseline association between IgM anti-MDA-LDL antibodies and size of the NC is consistent with these antibodies being directly involved in modifying plaque biology. However, although the inverse association between IgM anti-MDA-LDL antibodies and NC percentage survived adjustment for total serum IgM, other IgM antibodies are no doubt also involved. Firstly, there was a significant link between total serum IgM and NC percentage. Secondly, adjustment for total serum IgM attenuated the inverse association between IgM anti-MDA-LDL antibodies and NC volume and LCBI measured by RF-IVUS and NIRS respectively. Thirdly, in vitro and preclinical mouse studies indicate a broad role for IgM antibodies in facilitating safe clearance of debris of various types, including modified lipoproteins, apoptotic cells, cholesterol crystals and microparticles [3, 34].

In contrast to IgM anti-MDA antibodies and total serum IgM, IgG anti-MDA-LDL antibodies and total serum IgG did not relate to plaque characteristics. In previous work we demonstrated a strong inverse relationship of total serum IgG with major adverse coronary outcomes in the primary prevention hypertensive Anglo Scandinavian Cardiac Outcomes Trial (ASCOT) population [7]. However, it is notable that the interquartile range of total serum IgG levels in our IBIS-3 patients (8.3–13.1 g/L) is within the expected normal laboratory range, corresponding to the highest risk bottom tertile of values in the ASCOT patients (<13.1 g/L). This indicates that the populations are quite different, and highlights how distinct ‘at risk’ primary populations may be from those with established coronary disease. The current findings are consistent with a model in which IgM antibodies directly influence “vulnerable plaque”, whilst IgG provides useful insight into the “vulnerable patient” by reflecting a general systemic role(s). This role may perhaps be having a positive effect by protecting from systemic infections and infection-related inflammatory drive to atherosclerosis and cardiovascular events [35, 36]. Interestingly, there is also now evidence that (un)switched memory B cells have a protective effect on secondary cardiovascular events [37].

As was the case in the core IBIS-3 study [23], our sub-study failed to show retardation of total plaque burden progression over the course of a year, although there was a significant reduction in percentage of NC within lesions. However, a limitation of the study is that we do not know how plaques would have progressed in the absence of rosuvastatin. Moreover, we do not know whether low immunoglobulin or specific antibody levels would have predicted plaque development and changes in characteristics of vulnerability without the confounding influence of statin treatment. It is interesting that IgM levels dropped over the course of the year, and whether this can be attributed to statin treatment or even an effect of aging remains to be determined. One further limitation of this study, as well as previous studies in the field, is that the antigen used is laboratory modified MDA-LDL that will express other epitopes apart from MDA-modified protein. This polyclonal reactivity against a spectrum of modified LDL, or what is more commonly known as oxLDL, is challenging to dissect and has always been a limitation of studies using any form of laboratory-modified LDL as an antigen [38].

The clinical translational value of our findings, linking antibody levels to both favorable clinical outcomes as well as favorable plaque characteristics, would be in utilization as part of focused patient selection strategies in clinical trials for novel agents that target high risk populations [39, 40], as well as in exploring avenues for targeted immunotherapies in atherosclerosis [41, 42].

## 5 Conclusion

We confirm in our study that IgM anti-MDA-LDL antibodies confer protection from the development of clinical CHD, demonstrated in a hypertensive population. Importantly, we have shown for the first time that low levels of IgM anti-MDA-LDL antibodies, and to a lesser extent total serum IgM, predict worse NC and LCBI characteristics assessed directly by intracoronary imaging. These observations not only provide much needed clinical support for the protective role of humoral immunity in atherosclerosis proposed by preclinical studies, but also point to the possible use of low IgM anti-MDA-LDL antibody levels as a surrogate marker of unfavorable plaque characteristics.

## Data sharing statement

All publically available data is in the article and supplementary files.

## Appendix A Supplementary data

Supplementary material Image 1
