# Supplementary material for: IgM anti-malondialdehyde low density lipoprotein antibody levels indicate coronary heart disease and necrotic core characteristics in the Nordic Diltiazem (NORDIL) study and the Integrated Imaging and Biomarker Study 3 (IBIS-3)
Source: eBioMedicine. 2018 Aug 18;36:63–72. doi: 10.1016/j.ebiom.2018.08.023 (PMC6197783; doi:10.1016/j.ebiom.2018.08.023)
Supplement: Supplementary file 1 — Supplementary material [file mmc1.docx]

**SUPPLEMENTARY METHODS**

**NORDIL sub-study**

The study recruited from 1992-1999 across 1032 health centers in Norway and Sweden, with mean 4.5-year follow-up. 10,881 patients were randomized and undertook the study, of which 1,988 were extracted for analysis. The samples were further sub-categorized by those that developed coronary heart disease (CHD) or cardiovascular disease (CVD) over the course of the study. Patients with a previous history of CVD at enrollment were excluded. CHD is defined as patients with fatal myocardial infarction (MI), non-fatal MI Q-wave criterium, non-fatal MI T-wave criterium, sudden death, new-onset ischemic heart disease or new-onset congestive heart failure. CVD cases are defined as patients with CHD, plus: other death due to CVD, death in acute cerebrovascular disease, subarachnoid bleeding, ischemic stroke,

intracerebral bleeding, unspecified cerebrovascular disease or surgical revascularization.

**Statistical Methods**

***NORDIL sub-study***

Non-normally distributed data were log-transformed. A crude comparison between cases and controls with regard to baseline characteristics was explored by t-tests, χ² tests or Wilcoxon–Mann–Whitney test. Age- and sex-adjusted partial correlation coefficients were computed to evaluate the correlation amongst the log_e_ transformed baseline antibodies.

The association between each antibody variable of interest and the risk of subsequent CVD events was reported as an odds ratio (OR) obtained from a conditional logistic regression model, first by treating the baseline exposure variable as a continuous variable, giving an odd of having an event per 1 SD increase with 95% confidence intervals (CIs) and secondly, by categorizing exposure variable into tertiles with the lowest tertile as reference. For each analysis, two regression models were performed on the analyses on predictive values of baseline antibody. The models were: model 1- unadjusted; model 2: adjusted for current smoking status, diabetes mellitus and baseline HDL. In order to assess the effect of adjustment for serum Ig, we added the paired antibody levels and serum Ig (ie IgM anti-MDA-LDL with IgM and IgG anti-MDA-LDL with IgG) to Model 2 (we called this Model 3). Collinearity was tested using the variance inflation factor.

***IBIS-3 sub-study***

Differences between those from the total cohort that were and were not included in our sub-cohort were tested using Chi-square test and Fisher’s Exact test for categorical variable and Student T-tests and Mann–Whitney U tests for normally and non-normally distributed continuous variables respectively. Since the amount of measured plaque and NC volume is, amongst others, dependent on the length of the measured segment, we standardized for segment length by dividing volume through segment length, followed by a multiplication by the median segment length. Changes in RF-IVUS endpoints were computed by follow-up minus baseline values. Thus, negative values represent a decrease over time .

Our further data analysis methods are in line with the original IBIS-3 study (21), including linear mixed models to test for differences between baseline and follow-up measurements and trend tests to assess the associations between the quartiles of the antibodies and the LCBI and RF-IVUS parameters in linear regression models. To ensure that the assumptions of the linear regression models were met, baseline plaque volume was logarithmically transformed while NC volume was transformed using a Box-Cox power transformation. Since these transformations compromise the interpretability of the betas from the regression models, we chose to report the absolute values of LCBI and RF-IVUS parameters (change) for each immunoglobulin quartile together with the p value for the association derived from a linear trend test.

As was the case in the original IBIS-3 study, all models were adjusted for age, sex, diabetes, smoking and previous use of statins. In a second model, antibodies/immunoglobulins were also adjusted for levels of LDL- and HDL-cholesterol. In a third model IgM/IgG antibody levels were additionally adjusted for total IgM/IgG levels. We first examined baseline antibodies in relation to baseline LCBI, NC, plaque burden and plaque volume. Subsequently, we examined baseline antibodies in relation to the change in repeated NIRS and RF-IVUS measurements with an additional adjustment for time between catheterizations.

**SUPPLEMENTARY TABLES**

**Supplementary Table 1. Baseline characteristics of the NORDIL sub-study population**

| Variable | Controls  (n=494) | Cases  (n=185) | p_value |
| --- | --- | --- | --- |
| Male | 293 (59.3%) | 114 (61.6%) | 0.58 |
| Age (Years) | 60.79 (6.37) | 61.25 (6.30) | 0.40 |
| Smokers | 94 (19.0%) | 65 (35.1%) | <0.0001 |
| SBP (mmHg) | 173.24 (19.47) | 174.16 (19.64) | 0.59 |
| DBP (mmHg) | 103.42 (6.97) | 103.40 (8.16) | 0.97 |
| BMI (Kg/m^2^ ) | 28.07 (4.37) | 28.13 (4.40) | 0.86 |
| Total Cholesterol (mmol/L) | 6.30 (1.09) | 6.36 (1.06) | 0.50 |
| LDL-Cholesterol (mmol/L) | 4.10 (1.04) | 4.26 (0.98) | 0.09 |
| HDL-Cholesterol (mmol/L) | 1.38 (0.54) | 1.30 (0.34) | 0.04 |
| Triglyceride (mmol/L)^¶^ | 1.50 (1.10, 2.10) | 1.60 (1.20, 2.10) | 0.30 |
| Glucose (mmol/L) ^¶^ | 5.05 (4.50, 5.70) | 5.10 (4.50, 5.90) | 0.31 |
| Creatinine (mmol/L) | 87.87 (13.47) | 89.48 (18.36) | 0.21 |
| Diabetes | 36 (7.3%) | 24 (13.0%) | 0.021 |
| Diltiazem | 235 (47.6%) | 86 (46.5%) | 0.80 |

**Legend:** Values are mean (standard deviation) or n (%). ¶Values are presented in median (interquartile range), p values by Wilcoxon–Mann–Whitney test.

SBP: systolic blood pressure; DBP: diastolic blood pressure; BMI: body mass index; LDL: low density lipoprotein; HDL: high-density lipoprotein

**Supplementary Table 2. Median and interquartile range values of antibodies at baseline in Cardiovascular Disease cases versus controls in the NORDIL sub-study**

| Variable | Controls  (n=494) | Cases  (n=185) | p value |
| --- | --- | --- | --- |
| IgG anti-MDA-LDL (U) | 1.02 (0.83, 1.22) | 1.06 (0.84, 1.32) | 0.38 |
| IgM anti-MDA-LDL (U) | 0.85 (0.55, 1.20) | 0.76 (0.54, 1.04) | 0.039 |

**Legend:**

P values (interquartile range) obtained by Wilcoxon–Mann–Whitney test. Ig: immunoglobulin; MDA-LDL: malondialdehyde-modified low density lipoprotein; U: units

**Supplementary Table 3. Associations between baseline IVUS and NIRS parameters and baseline oxidized low density lipoprotein and Apolipoprotein-B in the IBIS-3 sub-study**

|  |  | Plaque Volume | Plaque Burden | NC Volume | NC Percentage | LCBImax4mm |
| --- | --- | --- | --- | --- | --- | --- |
| Biomarker |  | Median mm3 (IQR) | Median % (IQR) | Median mm3 (IQR) | Median % (IQR) | Median score (IQR) |
| Oxidized LDL | Lowest | 212.6 (118.5, 268.7) | 41.7 (32.1, 47.2) | 20.3 (5.1, 30.9) | 22.0 (18.2, 23.6) | 254.0 (83.0, 354.5) |
|  | Second | 231.7 (140.4, 332.0) | 40.5 (30.6, 47.2) | 21.5 (7.8, 57.3) | 19.7 (14.8, 27.3) | 179.0 (57.0, 320.8) |
|  | Third | 196.9 (167.5, 276.4) | 40.3 (33.5, 48.8) | 18.6 (7.2, 27.8) | 19.5 (15.8, 25.0) | 171.0 (83.0, 329.0) |
|  | Highest | 213.1 (151.4, 327.2) | 43.0 (34.7, 47.8) | 17.7 (9.2, 34.9) | 19.5 (15.7, 23.2) | 133.0 (27.5, 293.0) |
| Trend Model |  | p = 0.69 | p = 0.45 | p = 0.85 | p = 0.44 | p = 0.19 |
|  |  |  |  |  |  |  |
| ApoB | Lowest | 225.5 (147.8, 300.4) | 42.3 (32.5, 46.8) | 24.7 (6.6, 36.7) | 19.9 (15.4, 22.9) | 219.0 (65.5, 347.0) |
|  | Second | 229.6 (149.7, 316.0) | 43.1 (33.4, 48.4) | 22.5 (8.9, 37.3) | 20.9 (17.5, 25.4) | 183.5 (92.8, 332.5) |
|  | Third | 212.6 (186.1, 332.0) | 41.5 (33.5, 47.4) | 20.1 (14.2, 45.1) | 22.0 (18.6, 24.9) | 228.5 (31.3, 328.2) |
|  | Highest | 180.5 (137.3, 235.1) | 37.6 (31.9, 49.1) | 12.8 (6.2, 22.8) | 17.7 (11.8, 23.4) | 142.5 (30.5, 294.5) |
| Trend Model |  | p = 0.22 | p = 0.61 | p = 0.27 | p = 0.14 | p = 0.22 |

**Legend:**

P values based on a linear trend test across the four quartiles of the antibodies in a linear regression model, adjusted for age, sex, diabetes, smoking, and previous use of statins. RF-IVUS volumes are standardized for the measured segment length by dividing volume through segment length and then multiplication by the median segment length. mm^3^: cubic millimeter; IQR: interquartile range; NC: necrotic core tissue; LCBI: lipid core burden index; LDL: low density lipoprotein; ApoB: Apolipoprotein B

Limits of immunoglobulin and specific antibody quartiles are as in Table 3.

**Supplementary Table 4. Change in RF-IVUS measurements per quartile of baseline antibodies in the IBIS-3 sub-study**

|  |  | Plaque Volume | Plaque Burden | NC Volume | NC percentage |
| --- | --- | --- | --- | --- | --- |
| Biomarker |  | Median mm3 (IQR) | Median % (IQR) | Median mm3 (IQR) | Median % (IQR) |
|  |  |  |  |  |  |
| IgM anti-MDA- LDL antibodies | Lowest | 5.81 (-8.73, 20.89) | 0.29 (-1.88, 2.50) | -0.91 (-7.07, 3.47) | -0.96 (-3.27, 1.58) |
|  | Second | 0.46 (-9.94, 14.36) | 0.53 (-2.04, 3.51) | -1.64 (-8.82, 1.29) | -2.87 (-4.45, 0.63) |
|  | Third | -1.00 (-13.61, 14.07) | 1.11 (-0.27, 2.72) | 0.13 (-3.07, 2.68) | 0.67 (2.00, 2.06) |
|  | Highest | 6.34 (-5.86, 17.44) | 1.36 (-0.62, 3.35) | -0.42 (-2.81, 1.53) | -1.07 (-5.08, 0.62) |
| Trend Model 1 |  | p = 0.21 | p = 0.058 | p = 0.18 | p = 0.23 |
| Trend Model 2 |  | p = 0.56 | p = 0.08 | p = 0.55 | p = 0.52 |
|  |  |  |  |  |  |
| Total Serum IgM | Lowest | 2.66 (-11.41, 19.80) | 0.29 (-1.60, 2.96) | -0.73 (-7.64, 1.29) | -0.82 (3.30, 1.04) |
|  | Second | 0.59 (-6.48, 14.87) | 0.72 (-1.98, 2.28) | -0.34 (-5.61, 2.60) | -1.40 (4.25, 1.82) |
|  | Third | 6.12 (-5.93, 16.37) | 1.11 (-0.84, 3.11) | -0.41 (-3.07, 1.42) | -0.97 (-4.34, 1.68) |
|  | Highest | 4.96 (-9.23, 15.36) | 1.45 (-0.31, 3.48) | 0.063 (3.32, 2.23) | -0.54 (-3.98, 2.49) |
| Trend Model 1 |  | p = 0.22 | p = 0.39 | p = 0.17 | p = 0.28 |
|  |  |  |  |  |  |
| IgG anti-MDA- LDL antibodies | Lowest | 7.52 (-3.92, 21.25) | 1.25 (-0.23, 4.97) | -0.63 (-5.07, 1.43) | -1.38 (-4.51, 0.08) |
|  | Second | 9.05 (-6.14, 17.41) | 1.41 (-1.09, 2.41) | 0.58 (-1.54, 4.67) | 0.47 (-2.15, 2.37) |
|  | Third | -1.00 (-9.37, 8.35) | -0.18 (1.44, 1.61) | -1.69 (-5.05, 0.00) | -1.76 (-4.90, 1.66) |
|  | Highest | 5.45 (-13.36, 20.12) | 0.78 (-2.16, 3.28) | -0.37 (-4.88, 1.86) | -1.00 (-4.29, 1.59) |
| Trend Model 1 |  | p = 0.93 | p = 0.10 | p = 0.70 | p = 0.28 |
| Trend Model 2 |  | p = 0.70 | p = 0.18 | p = 0.95 | p = 0.28 |
|  |  |  |  |  |  |
| Total Serum IgG | Lowest | 4.44 (-4.68, 21.25) | 1.42 (-0.38, 3.37) | -0.46 (-4.15, 3.09) | -0.54 (-4.20, 2.18) |
|  | Second | 8.67 (-5.52, 16.58) | 0.92 (-0.61, 3.48) | 0.06 (-2.00, 3.91) | -1.07 (4.32, 2.36) |
|  | Third | -8.14 (-21.62, 12.16) | -0.41 (-2.51, 1.67) | -3.25 (-8.10, 0.35) | -2.23 (-5.34, -0.42) |
|  | Highest | 4.47 (-5.77, 18.10) | 1.26 (-0.78, 3.36) | -0.04 (-1.98, 1.80) | 0.26 (-1.59, 1.69) |
| Trend Model 1 |  | p = 0.19 | p = 0.27 | p = 0.20 | p = 0.88 |

**Legend**

P values based on a linear trend test across the four quartiles of the antibodies in a linear regression model.

**Model 1:** adjustment for age, sex, diabetes, smoking, LDL and HDL-cholesterol and previous use of statins and time to re-catherisation.

**Model 2:** Model 1 plus either total IgG or IgM

RF-IVUS volumes are standardized for the measured segment length by dividing volume through segment length and then multiplication by the median segment length. IQR: interquartile range; mm^3^: cubic millimeter; Ig: immunoglobulins; NC: necrotic core; MDA-LDL: Malondialdehyde-modified low density lipoprotein

**Supplementary Table 5. Change in NIRS measurements per quartile of baseline antibodies in the IBIS-3 sub-study**

|  |  | LCBI full region of interest | LCBI max 10mm | LCBI max 4mm |
| --- | --- | --- | --- | --- |
| Biomarker |  | Median score (IQR) | Median score (IQR) | Median score (IQR) |
|  |  |  |  |  |
| IgM anti-MDA- LDL antibodies | Lowest | -1.0 (-30.0, 35.5) | -29.0 (-79.7, 37.3) | -37.0 (-130.2, 27.5) |
|  | Second | 10.0 (-1.5, 20.0) | 9.0 (-19.5, 58.3) | 19.0 (-26.0, 98.8) |
|  | Third | -1.0 (-24.8, 18.0) | 5.0 (-44.3, 59.8) | 30.5 (-57.0, 83.8) |
|  | Highest | 8.5 (-0.8, 24.5) | 5.5 (-41.3, 79.8) | 43.5 (1.3, 99.8) |
| Trend |  | p = 0.750 | p = 0.285 | p = 0.145 |
|  |  |  |  |  |
| Total Serum IgM | Lowest | 3.0 (-16.5, 35.5) | -22.0 (-63.0, 71.0) | -27.0 (-75.5, 60.5) |
|  | Second | 0.0 (-13.8, 12.5) | -3.0 (-34.0, 15.0) | 15.0 (-79.0, 41.0) |
|  | Third | 6.5 (-2.0, 30.0) | 36.0 (0.0, 64.5) | 37.5 (-43.3, 107.0) |
|  | Highest | 4.0 (-19.5, 22.8) | 2.5 (-52.5, 69.3) | 29.0 (0.5, 93.8) |
| Trend |  | p = 0.946 | p = 0.325 | p = 0.223 |
|  |  |  |  |  |
| IgG anti-MDA- LDL antibodies | Lowest | 1.0 (-14.5, 20.0) | -6.0 (-69.0, 26.8) | -8.5 (94.8, 37.0) |
|  | Second | 12.5 (-14.5, 24.3) | -2.0 (-30.5, 78.3) | 23.5 (-44.8, 108.8) |
|  | Third | 2.0 (-8.5, 42.3) | 24.5 (-15.0, 79.3) | 45.0 (-38.3, 148.8) |
|  | Highest | 3.5 (-14.0, 14.8) | 1.5 (52.5, 45.5) | 10.0 (-43.3, 61.3) |
| Trend |  | p = 0.841 | p = 0.472 | p = 0.268 |
|  |  |  |  |  |
| Total Serum IgG | Lowest | 9.0 (-6.5, 28.5) | 15.0 (-19.5, 82.5) | 41.0 (1.5, 103.0) |
|  | Second | 5.0 (-17.3, 23.8) | -4.0 (-66.8, 11.0) | 8.0 (-64.8, 50.5) |
|  | Third | 4.0 (-10.8, 20.3) | 16.0 (-50.0, 62.3) | 9.5 (-80.3, 95.5) |
|  | Highest | -0.5 (-36.0, 24.0) | 0.0 (-53.0, 63.0) | 0.0 (-59.0, 65.0) |
| Trend |  | p = 0.302 | p = 0.413 | p = 0.342 |

**Legend**

P values based on a linear trend test across the four quartiles of the antibodies in a linear regression model, with adjustment for age, sex, diabetes, smoking, and previous use of statins.

NIRS: Near-Infrared Spectroscopy; IQR: interquartile range; NC: necrotic core tissue; Ig: immunoglobulins; MDA-LDL: Malondialdehyde-modified low density lipoprotein; HDL: high-density lipoprotein
